# Description and Genomic Characterization of *Oceaniferula flavus* sp. nov., a Novel Potential Polysaccharide-Degrading Candidate of the Difficult-to-Cultivate Phylum Verrucomicrobiota Isolated from Seaweed

**DOI:** 10.3390/md21010031

**Published:** 2022-12-29

**Authors:** Meng-Qi Ye, Chuan-Bo Jin, Xin-Jiang Liu, Xin-Yun Tan, Yu-Qi Ye, Zong-Jun Du

**Affiliations:** 1 Marine College, Shandong University, Weihai 264209, China; 2 Weihai Research Institute of Industrial Technology, Shandong University, Weihai 264209, China; 3 State Key Laboratory of Microbial Technology, Shandong University, Qingdao 266237, China

**Keywords:** *Oceaniferula flavus*, difficult-to-culture microorganism, genomic analysis, phylogenetic analysis, polysaccharide degrading

## Abstract

A novel strain, isolate 5K15^T^, which belongs to difficult-to-cultivate phylum *Verrucomicrobiota*, was recovered from kelp collected from Li Island, Rongcheng, China. The genome sequence of the strain (genome size 3.95 Mbp) showed the presence of four putative biosynthetic gene clusters (BGCs), namely, two terpene biosynthetic gene clusters, one aryl polyene biosynthetic cluster, and one type III PKS cluster. Genomic analysis revealed 79 sulfatase-encoded genes, 24 sulfatase-like hydrolase/transferase-encoded genes, and 25 arylsulfatase-encoded genes, which indicated the great potential of 5K15^T^ to degrade sulfated polysaccharides. Comparative analysis of 16S rRNA gene sequence showed that the novel strain was most closely related to *Oceaniferula marina* N1E253^T^ (96.4%). On the basis of evidence from a polyphasic study, it is proposed that the strain 5K15^T^ (= KCTC 82748^T^ = MCCC 1H00442^T^ = SDUM 810003^T^) be classified as *Oceaniferula flavus* sp. nov. The strain has the ability of carbohydrate transport and metabolism. This ability allows it to survive in carbohydrate-rich materials such as kelp. It has the potential to be used in the marine drug industry using seaweed.

## 1. Introduction

Seaweed is an abundant resource in the ocean. It contains functional components such as polysaccharides, polyunsaturated fatty acids, proteins, fiber, vitamins, amino acids, peptides, lipids, and minerals [1,2,3,4] that have various applications. Edible seaweed represents a bountiful marine drug source with health advantages, such as reducing the risk of chronic diseases [5,6,7]. The consumption of seaweed has become increasingly popular. In addition to direct consumption, a significant portion of harvested seaweed is utilized for processed food or in manufacturing of active substances or drugs with remarkable biological properties, such as polysaccharides carrageenan, agar, alginate, and some oligosaccharides [5]. There is increasing interest in using seaweed for global food security and other products, and thus the global demand for seaweed has grown exponentially over the last 25 years [8].

In the wake of the attention attracted by seaweed, the seaweed-associated microbes that form functional symbionts with seaweed also are of interest [1,9]. The surface of seaweed supports an abundant diverse microbiota. The interplay between seaweed and its microbiome can affect seaweed in diverse ways, such as in the growth, the defense, or destruction mechanisms, especially in terms of the degradation of seaweed and the production of biologically active metabolites [10,11,12]. Strains that can degrade seaweed efficiently are highly important in the development of marine algae drugs.

During our previous studies of seaweed microbial populations, a number of bacteria were cultured, including a novel strain 5k15^T^. In this study, this new taxon was classified with polyphasic taxonomy, including biochemical, chemotaxonomic, and phylogenetic analysis methods. The polyphasic evidence indicated that the strain 5K15^T^ represents a novel phylogenetic species of the genus *Oceaniferula*. The genus *Oceaniferula* was first described by Jin et al. in 2021 [13] and contains only one recognized species, *Oceaniferula marina*, up to now. The type strain of *O. marina* was N1E253^T^*,* which was isolated from marine sediment. N1E253^T^ possesses a draft genome size of 5.07 Mbp. The strain has an assimilatory sulfate reduction pathway that can assimilate sulfate into hydrogen sulfide. It also can dissimilate nitrate into nitrite. Most of the coding sequences in the genome were classified as unknown function, indicating that this genus has a lot of potentially unknown features. The genus *Oceaniferula* belongs to the family *Verrucomicrobiaceae* order *Verrucomicrobiales* class *Verrucomicrobiae*, phylum *Verrucomicrobiota*. Phylum *Verrucomicrobiota* is a core group of the PVC superphylum (*Planctomycetes*–*Verrucomicrobiota*–*Chlamydiae*, as well as *Lentisphaerae*, *Kirimatiellaeota,* and some uncultured candidate phyla) [14]. Although the phylum *Verrucomicrobiota* was reported to be ubiquitous in different habitats, it belongs to difficult-to-culture bacteria. They are important and unusual from many perspectives [15], especially in terms of their brilliant potential to degrade the sulfated polysaccharides, which are an important component of seaweed [16,17]. The sulfated polysaccharides in seaweeds such as fucoidans are probably recalcitrant and more difficult to degrade. No sufficient and convenient sources with enzyme activity are available yet for the degradation of fucalean algae. The abundance of *Verrucomicrobiota* was even coupled with the algae bloom onset [17]. In our previous study, we also found that *Verrucomicrobiota* was highly abundant in kelp. This indicated that *Verrucomicrobiota* is a potential source for degrading various sulfated polysaccharides. 

The strain 5k15^T^ in this study is a new member of the genus *Oceaniferula* phylum *Verrucomicrobiota* isolated from the seaweed habitat. The present study was designed to determine the taxonomic status, ecological characteristics, and potential application of the *Oceaniferula* strain, isolate 5K15^T^, recovered from kelp collected from Li Island, RongCheng, China. 

## 2. Results and Discussion

### 2.1. Isolation and Maintenance of Strain 5K15^T^

Strain 5K15^T^, showing a yellow-colored, circular, and smooth colony, was isolated from kelp cultivated on Li Island, Rongcheng, China (37.2661° N, 122.6138° E). The purified culture was stored at −80 °C in sterile 15% (*v/v*) glycerol supplemented with 1% (*w/v*) NaCl. *Oceaniferula marina* N1E253^T^ (= KCTC 72800^T^ = MCCC 1H00405^T^) was used as a related type strain for the comparison of phenotypic and chemotaxonomic characteristics.

### 2.2. 16S rRNA Gene Sequence and Phylogenetic Analysis

**Figure 1:**
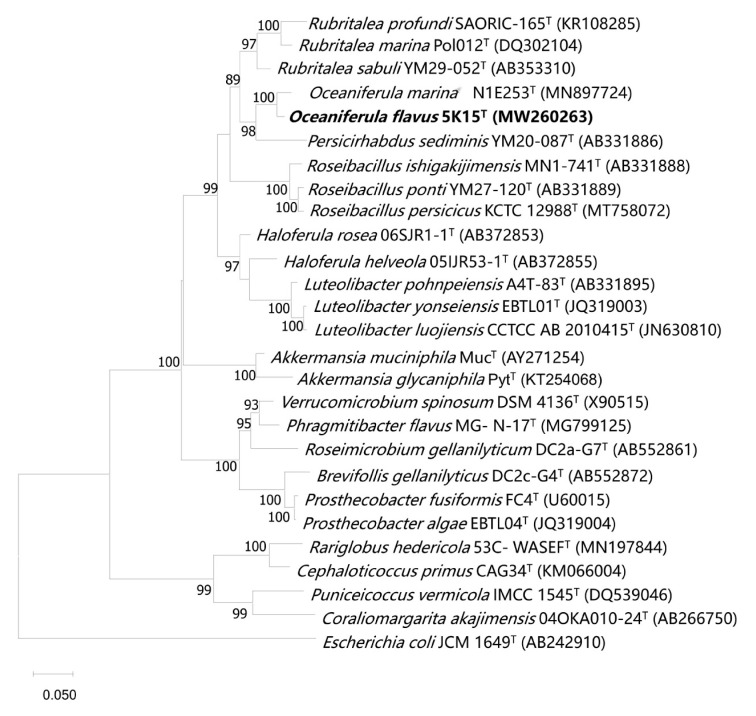
Maximum likelihood phylogenetic tree based on 16S rRNA gene sequences showing the phylogenetic position of strain 5K15^T^ among closely related taxa. Bootstrap values (expressed as percentages of 1000 replications) of >70% are shown at branch points. *Escherichia coli* JCM 1649^T^ (AB242910) was used as an outgroup. Bar, 0.05 nucleotide substitutions per position.

Analysis of the 16S rRNA gene sequences (1462 bp) clearly showed that the most closely related species to strain 5K15^T^ was *O. marina* N1E253^T^ (96.4%), followed by *Persicirhabdus sediminis* KCTC 22039^T^ (91.9%), *Rubritalea sabuli* KCTC 22127^T^ (91.8%), and *R. profundi* KCTC 52460^T^ (91.7%). The topological structure of the phylogenetic maximum likelihood tree (Figure 1) clearly illustrated that strain 5K15^T^ was affiliated to the genus *Oceaniferula*, supported by a high bootstrap value of 100%. The tree topology was also supported by FastTree and IQTree (Figures S1 and S2). Therefore, it would appear that, on the basis of the sequence identity and phylogenetic data, strain 5K15^T^ may represent a novel species of the genus *Oceaniferula*.

### 2.3. Morphological, Physiological, and Biochemical Characteristics

Cells of 5K15^T^ were Gram-stain-negative, facultatively anaerobic, non-flagellated, non-motile, rod-shaped, and approximately 0.3–0.5 µm in width and 0.7–2.0 µm in length (Figure S3). Gliding motility was not observed. Growth occurred at 15–37 °C (optimum, 33 °C), pH 6.0–8.5 (optimum, 7.5), and in the presence of 1.0–5.5% (*w/v*) NaCl (optimum, 2.0–3.5%). No growth occurred at pH 5.5 or pH 9.0. It was positive for nitrate reduction, but negative for catalase, oxidase, DNase, and hydrolysis of starch; casein; cellulose; and Tweens 20, 40, 60, and 80. Strain 5K15^T^ was susceptible to ceftriaxone (30 µg), cefotaxime (30 µg), ampicillin (10 µg), carbenicillin (100 µg), penicillin (30 µg), and lincomycin (2 µg), but resistant to rifampin (5 µg), chloramphenicol (30 µg), clarithromycin (15 µg), erythromycin (15 µg), ofloxacin (5 µg), gentamycin (10 µg), tetracycline (30 µg), kanamycin (30 µg), tobramycin (10 µg), vancomycin (30 µg), neomycin (30 µg), norfloxacin (10 µg), and streptomycin (10 µg). 

The strain was found to be positive for the Voges–Proskauer reaction, but negative for citrate utilization; arginine dihydrolase; lysine decarboxylase; ornithine decarboxylase; urease; tryptophan deaminase; and gelatinase, H_2_S, and indole production. Activities of esterase (C4), leucine arylamidase, valine arylamidase, acid phosphatase, and naphthol-AS-BI-phosphphydrolase were present, but activities of alkaline phosphatase, esterase lipase (C8), lipase (C14), cystine arylamidase, trypsin, α-chymotrypsin, α-galactosidase, β-galactosidase, β-glucuronidase, α-glucosidase, β-glucosidase, N-acetyl-β-glucosaminidase, α-mannosidase, and β-fucosidase were absent. Acid was produced from d-ribose, d-xylose, d-fructose, l-sorbitose, d-lyxose, d-tagatose, and 5-ketogluconate. The following carbon sources were oxidized: d-raffinose, α-d-lactose, α-d-glucose, d-mannose, d-mannitol, d-fructose, d-galactose, l-lactic acid, d-cellobiose, gentiobiose, d-turanose, d-fructose-6-PO_4_, glucuronamie, and l-malic acid. Other physiological and biochemical characteristics of 5K15^T^ are shown in Table 1.

### 2.4. Chemotaxonomic Characterization

The sole respiratory quinone of strain 5K15^T^ was MK-9, which was similar to *O. marina* N1E253^T^. A list of the fatty acid compositions of strains 5K15^T^ and N1E253^T^ are detailed in Table 2. The fatty acid profile of strain 5K15^T^ was similar to that of *O. marina* N1E253^T^, although there were differences in the proportions of some components. The major fatty acids (>10% of the total) of strain 5K15^T^ were iso-C_14:0_ (28.5%), anteiso-C_15: 0_ (11.8%), and C_16:0_ (10.0%). The major polar lipids of strain 5K15^T^ were phosphatidylethanolamine (PE), diphosphatidylglycerol (DPG), and one unidentified phospholipid (PL). Minor amounts of phosphatidylglycerol (PG), one unidentified aminolipid (AL), and one unidentified glycolipid (GL) were also present. These polar lipids were also present in *O. marina* N1E253^T^ but with different contents. In addition, three unidentified polar lipids (L1, L2, L3) were only present in strain *O. marina* N1E253^T^. Detailed polar lipid images of different strains are represented in Figure S4. 

### 2.5. Genome insight of the Two Strains of Genus Oceaniferula

#### 2.5.1. Genome Properties

The draft genome sequences of strain 5K15^T^ were assembled into 51 contigs, and the total genome length was 3,953,077 bp, which was smaller than that of *O. marina* N1E253^T^ (5,073,947 bp) (Table 3). The sequencing depth of coverage was 363×. The genome DNA G+C content of strain 5K15^T^ was 55.4 mol%, which was higher than that of *O. marina* N1E253^T^ (52.0 mol%). 

**Figure 2:**
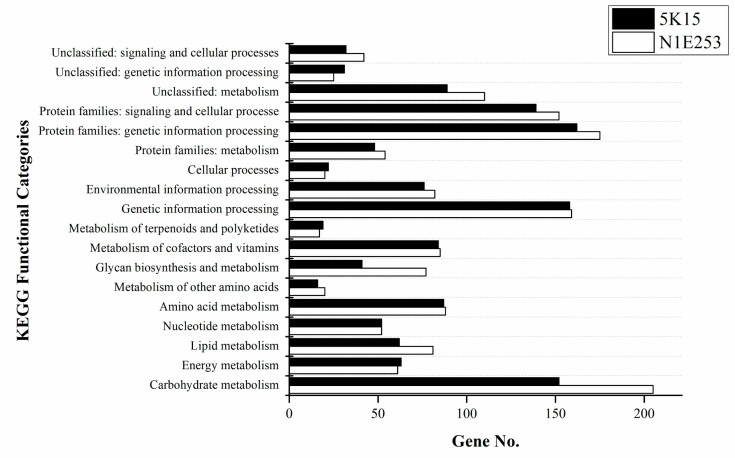
Bar diagram of annotated KEGG functional categories of strains 5K15^T^ and *O. marina* N1E253^T^.

The draft genome of 5K15^T^ was predicted to contain 3354 genes, including 3293 protein-coding genes, 45 tRNAs, 3 rRNAs, and 4 ncRNAs by the NCBI Prokaryotic Genome Annotation Pipeline server online (PGAP). According to annotations from the KEGG database, 1426 genes (43.3%) could be assigned a putative function, including genetic information processing (162 genes), carbohydrate metabolism (152 genes), signaling and cellular processes (139 genes), amino acid metabolism (87 genes), metabolism of cofactors and vitamins (84 genes), environmental information processing (76 genes), energy metabolism (63 genes), lipid metabolism (62 genes), glycan biosynthesis and metabolism (41 genes), and drug resistance (47 genes). The comparison of 5K15^T^ and *O. marina* N1E253^T^ is shown in Figure 2.

**Figure 3:**
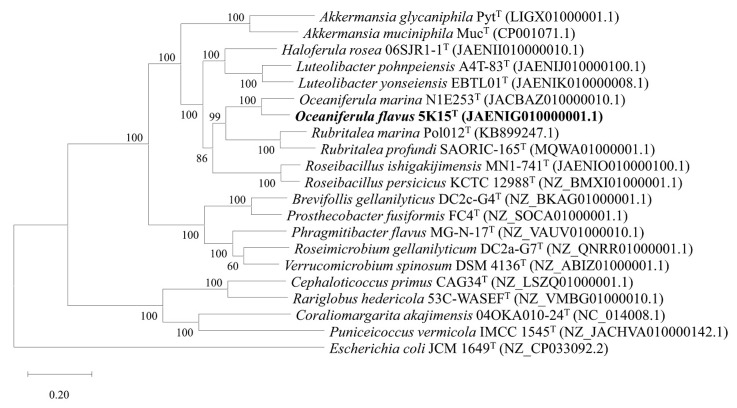
The IQ-TREE based on 120 ubiquitous single-copy genes. *Escherichia coli* JCM 1649^T^ was used as the outgroup. Bar, 0.20 substitutions per nucleotide position.

The ANI value between strain 5K15^T^ and *O. marina* N1E253^T^ was 77.1%. This value is below the threshold value (95.0%) for species delineation [18]. The dDDH value of strain 5K15^T^ in comparison to *O. marina* N1E253^T^ was lower than 20.0%. This value is much lower than the threshold for species demarcation (70.0%) [19]. The POCP value between strain 5K15^T^ and *O. marina* N1E253^T^ was 50.6%, which is higher than the genus boundary cut-off of 50% [20]. The phylogenetic tree based on a concatenated alignment of 120 ubiquitous single-copy genes showed the evolutionary relationships of strain 5K15^T^ and related strains (Figure 3). Combined with the 16S rRNA gene sequence identity, these data indicate that strain 5K15^T^ represents a novel species of the genus *Oceaniferula*. 

It is, therefore, proposed that isolate 5K15^T^ be considered as the type strain of a novel *Oceaniferula* species that belongs to the phylum *Verrucomicrobiota*. The name proposed for this type species is *Oceaniferula flavus* sp. nov.

#### 2.5.2. Metabolic Pathways

**Figure 4:**
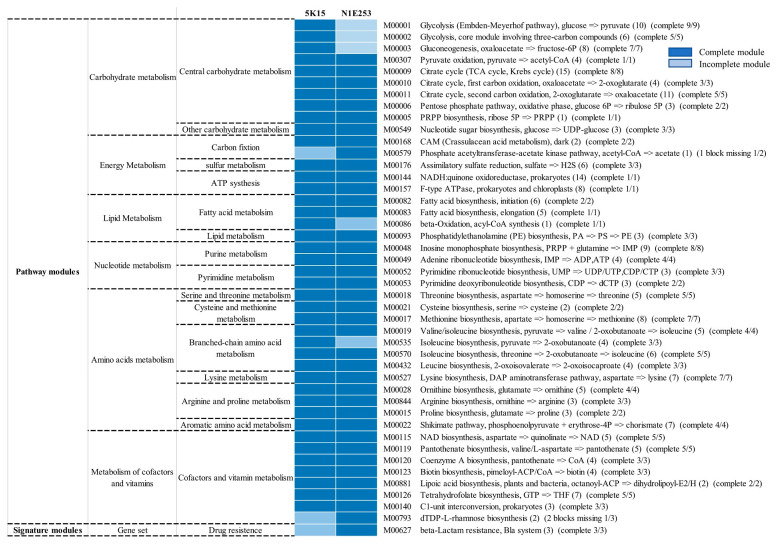
Heat maps of complete and incomplete metabolic pathways in the genomes of strains 5K15^T^ and *O. marina* N1E253^T^.

**Figure 5:**
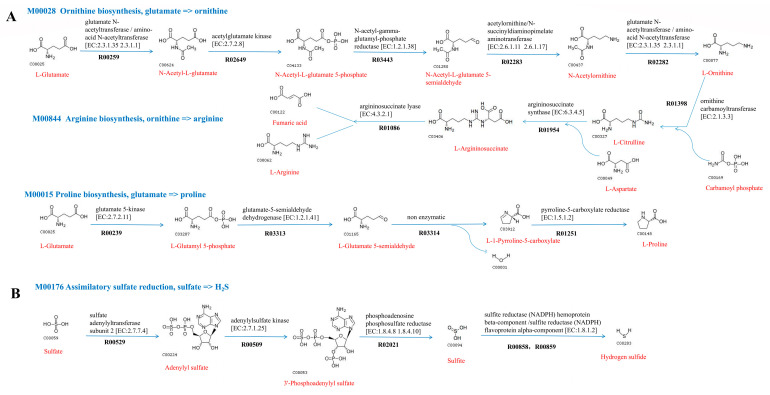
Amino acid metabolism (**A**) and assimilatory sulfate reduction pathway (**B**) of the genus *Oceaniferula*.

Analysis of metabolic pathways was performed using KEGG’s BlastKOALA service. Many complete metabolic modules were found in the genomes of strain 5K15^T^ and *O. marina* N1E253^T^, such as the complete central carbohydrate metabolism, energy metabolism, lipid metabolism, nucleotide metabolism, amino acid metabolism, and cofactor and vitamin metabolism (Figure 4). For example, the genomes of strain 5K15^T^ and *O. marina* N1E253^T^ both contain a complete set of genes of the arginine and proline metabolism pathway (M00028, M00844, and M00015) (Figure 5A). Both strains contain genes *argJ*, *argB*, *argC*, *argD,* and *argJ*, which support transformation from glutamate to ornithine. The presence of genes *argF*, *argG,* and *argH* allows them to use ornithine to synthesize arginine. The strain 5K15^T^ also has the ability to perform isoleucine biosynthesis through two pathways modules (M00535 and M00570), but only *O. marina* N1E253^T^ can biosynthesize isoleucine through one module (M00570). In addition, both *O. marina* N1E253^T^ and strain 5K15^T^ have genes *cysN*, *cysD*, *cysC*, *cysH*, *cysJ,* and *cysI*, which can assimilate sulfate into hydrogen sulfide (M00176) for energy metabolism (Figure 5B). As for other energy metabolism pathways, these two strains also possess a carbon fixation ability through CAM (Crassulacean acid metabolism) pathways (M00168). Only *O. marina* N1E253^T^ has a phosphate acetyltransferase-acetate kinase pathway (M00579), which is also a kind of carbon fixation pathway. For cofactor and vitamin metabolism, unlike *O. marina* N1E253^T^, strain 5K15^T^ lacks genes for glucose-1-phosphate thymidylyltransferase (EC 2.7.7.24) and dTDP-4-dehydrorhamnose reductase (EC 1.1.1.133); furthermore, the dTDP-L-rhamnose biosynthesis pathway was found to be incomplete (M00793). 

The functional annotation of the two strains were also conducted by searching against the COG database. Overall, except for the unknown function, the function of carbohydrate transport and metabolism, inorganic ion transport and metabolism, amino acid transport and metabolism, and cell wall/membrane/envelope biogenesis were the major functional categories (Figure S5). The ability of carbohydrate transport and metabolism makes the strains able to live in kelp, which is rich in carbohydrates. On the other hand, the decomposition and utilization of kelp carbohydrates can lead to the decay of fresh kelp, which is subversive for the cultivation of kelp. However, considering the deep processing of kelp, the decomposition and utilization of kelp by the bacteria can contribute to the production of kelp degradation products.

#### 2.5.3. Prediction of Secondary Metabolites

Secondary metabolite analysis was performed by antiSMASH. The genome of strain 5K15^T^ was found to have four putative biosynthetic gene clusters (BGCs), including two terpene biosynthetic gene clusters, one aryl polyene biosynthetic cluster, and one type III PKS (polyketide synthase) cluster (Table S1). Aryl polyenes are structurally similar to carotenoids but are biosynthesized by a PKS rather than a terpene synthase, and they have been predicted to be protective agents in response to photo-oxidative damage, just like carotenoids [21]. Type III PKSs have been found in bacteria and fungi, as well as plants. Type III PKSs in microorganisms are involved in the biosynthesis of some lipidic materials and diverse secondary metabolites, which have significant biological functions and/or important pharmaceutical activities [22]. The strain *O. marina* N1E253^T^ has two terpene biosynthetic clusters and one phosphonate cluster (Table S1). The potential ability of multiple BGCs may help the bacteria grow better in a marine environment [23]. 

#### 2.5.4. Estimation of Carbohydrate-Active Enzymes (CAZymes)

**Figure 6:**
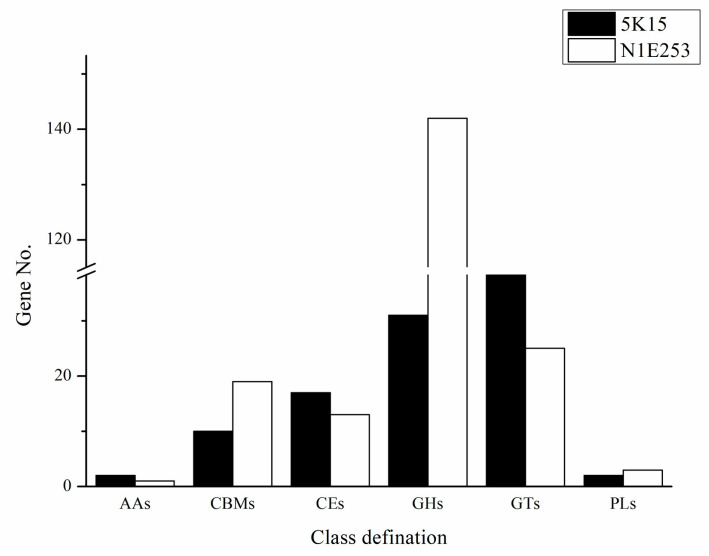
Histogram of predicted carbohydrate-active enzymes in strains 5K15^T^ and *O. marina* N1E253^T^. AAs, auxiliary activities; CBMs, carbohydrate-binding module; CEs, carbohydrate esterases; GHs, glycoside hydrolases; GTs, glycosyltransferases; PLs, polysaccharide lyases.

Considering the potential polysaccharide utilization ability of *Verrucomicrobiota*, the carbohydrate-active enzymes (CAZymes) of the two *Oceaniferula* genomes were estimated using the CAZy database. Strain 5K15^T^ and *O. marina* N1E253^T^ contained 106 and 203 carbohydrate-active enzymes, respectively (Figure 6). In the genome of strain *O. marina* N1E253^T^, more than 70% (142 enzymes) of the identified enzymes were assigned to the glycoside hydrolase (GH) family. Compared with *O. marina* N1E253^T^, glycosyltransferases (GT) are the predominant enzyme in the strain 5K15^T^. More than 41.5%, 29.2%, and 16.0% of the enzymes were assigned to the GT family (44 enzymes), GH family (31 enzymes), and carbohydrate esterases (17 enzymes) in the genome of strain 5K15^T^, respectively. The 31 glycoside hydrolases include fucosidases from the families GH29 and GH95, as well as fucoidanase from the families GH36, GH116, and GH117 with a yet unknown function and GH168 with activity of endo-1,3-fucanase. In addition, according to the NCBI Prokaryotic Genome Annotation Pipeline (PGAP) and the Database of Sulfatases (https://sulfatlas.sb-roscoff.fr/, accessed on 21 November 2022), the genome of strains 5K15^T^ and N1E253^T^ contained 79 and 137 sulfatase-encoded genes, 24 and 52 sulfatase-like hydrolase/transferase-encoded genes, and 25 and 36 arylsulfatase-encoded genes, respectively. The sulfatases of 5K15^T^ belonged to 15 families, including S1_15, S1_16, and S1_14. In the previous studies about the degrading ability of *Verrucomicrobiota,* genome annotation of strain *‘Lentimonas’* sp. CC4 revealed 100 glycoside hydrolases, 113 sulfatases, and 17 carbohydrate esterases [16]. *Verrucomicrobiota* MAGs carried, on average, ≈7 fucosidases per MAG [17], and the fucosidases of stain 5K15^T^ was above average. These results indicated that the genus *Oceaniferula* has great potential to degrade sulfated polysaccharides, which has not been noticed before due to rare cultivable strains to some extent.

### 2.6. Description of Oceaniferula flavus sp. nov. Oceaniferula flavus (L. masc. adj. flavus, Yellow, Referring to the Color of the Colonies)

Cells are Gram-stain-negative, facultatively anaerobic, rod-shaped and 0.3–0.5 µm wide, and 0.7–2.0 µm long. Neither cellular gliding movement nor mobile ability was observed. Colonies grown on 1/2-strength R2A agar with 75% artificial seawater were circular, convex, and yellow. Growth occurred at 15–37 ℃ (optimum, 33 ℃), pH 6.0–8.5 (optimum, 7.5), and 1.0–5.5% (*w/v*) NaCl (optimum, 2.0–3.5% (*w/v*)). It was positive for nitrate reduction, but negative for catalase, oxidase, and DNase, as well as hydrolysis of starch; casein; cellulose; and Tweens 20, 40, 60, and 80. It was positive for the Voges–Proskauer reaction, but negative for citrate utilization; arginine dihydrolase; lysine decarboxylase; ornithine decarboxylase; urease; tryptophan deaminase; and gelatinase, H_2_S, and indole production. Activities of esterase (C4), leucine arylamidase, valine arylamidase, acid phosphatase, and naphthol-AS-BI-phosphphydrolase were present, but activities of alkaline phosphatase, esterase lipase (C8), lipase (C14), cystine arylamidase, trypsin, α-chymotrypsin, α-galactosidase, β-galactosidase, β-glucuronidase, α-glucosidase, β-glucosidase, N-acetyl-β-glucosaminidase, α-mannosidase, and β-fucosidase were absent. Acid was produced from d-ribose, d-xylose, d-fructose, l-sorbitose, d-lyxose, d-tagatose, and 5-ketogluconate. The following carbon sources were oxidized: d-raffinose, *α*-d-lactose, *α*-d-glucose, d-mannose, d-mannitol, d-fructose, d-galactose, l-lactic acid, d-cellobiose, gentiobiose, d-turanose, d-fructose-6-PO_4_, glucuronamie, and l-malic acid. MK-9 was the sole respiratory quinone. The major fatty acids were iso-C_14:0_, anteiso-C_15:0_, and C_16:0_. The major cellular polar lipids were phosphatidylethanolamine, diphosphatidylglycerol, and one unidentified phospholipid.

The type strain was 5K15^T^ (= KCTC 82748^T^ = MCCC 1H00442^T^ = SDUM 810003^T^), isolated from kelp of Li Island, Rongcheng, China (37.2661° N, 122.6138° E). Genomic analysis indicated that the strain has great potential to degrade sulfated polysaccharides and can be applied in the marine drug industry.

## 3. Materials and Methods

### 3.1. Sample Collection, Isolation, and Preservation

In the process of screening for seaweed microbial populations, strain 5K15^T^ was isolated from kelp that was cultivated in Li Island, Rongcheng, China (37.2661° N, 122.6138° E). The fresh kelp sample was cut into small pieces and placed in sterile aged seawater followed by shaking at 300 rpm for 1 h. The 100 μL supernatant was spread on a self-made kelp powder medium (10 g kelp powder, 15 g agar, 1 L aged seawater). Plates were incubated in an aerobic environment at 30 °C for 7 days. A yellow-colored colony was isolated and further subcultivated on 1/2-strength R2A agar with 75% artificial seawater. The purified culture was stored at −80 ℃ in sterile 15% (*v/v*) glycerol supplemented with 1% (*w/v*) NaCl. *Oceaniferula marina* N1E253^T^ (= KCTC 72800^T^ = MCCC 1H00405^T^) was used as the related type strain for the comparison of phenotypic and chemotaxonomic characteristics.

### 3.2. Physiological and Biochemical Characteristics

The cell morphology and the presence of flagella and cell size were observed by using light microscopy (E600; Nikon) and scanning electron microscopy (model Nova NanoSEM450; FEI). The motility was tested with the hanging-drop method, and gliding motility was confirmed by inoculating the bacteria on the 0.5% agar, as described by Bowman [24]. Gram staining was conducted by using a Gram stain kit (bioMérieux). Growth at various temperatures (4, 10, 15, 20, 25, 28, 30, 33, 35, 37, 40, and 45 ℃) and at various NaCl concentrations (0–10%, at intervals of 0.5%) was tested on 1/2-strength R2A agar with 75% artificial seawater in triplicate. The pH range for growth was determined by adjusting the medium with 1 M HCl or NaOH, as well as the following buffers (Sangon, China): MES (pH 5.5 and 6.0), PIPES (pH 6.5 and 7.0), HEPES (pH 7.5 and 8.0), Tricine (pH 8.5), and CAPSO (pH 9.0, 9.5) with a concentration of 20 mM. Anaerobic growth was determined on 1/2-strength R2A agar with 75% artificial seawater with or without 0.1% (*w/v*) KNO_3_ incubated in an anaerobic jar (10% H_2_, 10% CO_2_, and 80% N_2_) for 14 days. Susceptibility to antibiotics was investigated using the disc-diffusion method as described previously [25]. The hydrolysis of starch, casein, alginate, carboxymethylcellulose, and Tweens (20, 40, 60, and 80) was investigated according to previously mentioned methods [26]. DNase activity was determined on DNase test agar (Hopebio, Qingdao, China). The catalase activity was determined by using the bubble formation in 3% H_2_O_2_ solution. The oxidase activity was tested with an oxidase reagent kit (bioMérieux, Lyon, France) according to the manufacturer’s instructions. The production of other enzymes was assessed using API ZYM (bioMérieux, Lyon, France) kits. Tests for acid production from different carbohydrates were performed using the API 50CHB system (bioMérieux, Lyon, France). Tests for the oxidation of carbon sources were conducted using the Biolog GEN III system. Tests for additional physiological and biochemical characteristics were conducted using the API 20E system (bioMérieux, Lyon, France). All the API and Biolog tests were carried out according to the manufacturer’s instructions, except that the concentration of the suspension medium was adjusted to 3.0% (*w/v*) NaCl. All tests were implemented simultaneously with strains 5K15^T^ and *O. marina* N1E253^T^ with two replicates.

### 3.3. Chemotaxonomy

The cell mass of strain 5K15^T^ was obtained from cultures grown for 3 days on 1/2-strength R2A agar with 75% artificial seawater at 33 °C. The biomass of strain *O. marina* N1E253^T^ was obtained from cultures grown on marine agar 2216 (MA) for 3 days at 30 °C. The fatty acids were saponified, methylated, and extracted according to the standard protocol of MIDI (Sherlock Microbial Identification System, version 6.1). Cellular fatty acids were analyzed using an Agilent 6890N gas chromatograph and identified using the TSBA40 database of the microbial identification system [27]. Respiratory quinones were extracted from 300 mg freeze-dried cells [28] and purified with a silica gel TLC plate (Merck Kieselgel 60 F254), then analyzed via HPLC (high-performance liquid chromatography) [29]. Polar lipids were determined using 2D thin-layer chromatography (TLC) [30].

### 3.4. DNA Sequencing and Phylogenetic Analyses

Universal bacterial primers 27F and 1492R [31] were used for the amplification of the 16S rRNA gene. The PCR product was purified and cloned into the pMD18-T vector (Takara, Dalian, China), and recombinant plasmids were reproduced in *Escherichia coli* DH5*α* cells (Trans-Gen Biotech, Beijing, China). Sequencing was performed by BGI Tech (Qingdao, China). To identify the taxonomic status of strain 5K15^T^, the cloned sequence was submitted to the GenBank databases, and sequence identities were calculated from alignments obtained by the BLAST algorithm (https://www.ncbi.nlm.nih.gov, accessed on 21 November 2020). The sequences of the novel strain and type strain were aligned by using MUSCLE (Edgar 2004). A phylogenetic tree was reconstructed by using the maximum-likelihood algorithm implemented in the software package MEGA X. Phylogenetic trees were also reconstructed by using FastTree [32] and IQTree [33]. FastTree and IQTree used the GTR+CAT parameters and the GTR+F+I+G4 model, respectively. The robustness of the phylogenetic trees was confirmed by performing a bootstrap analysis based on 1000 replications. 

### 3.5. Genomic DNA Sequencing, Assembly, Annotation, and Genome Comparison

The genomic DNA of strain 5K15^T^ was extracted using the bacterial genomic DNA Kit (Takara, Dalian, China). The draft whole-genome sequencing of the strain 5K15^T^ was carried out by Novogene (Beijing, China), using the Illumina Hiseq PE150 platform. All the good-quality sequenced reads were assembled using SOAPdenovo software into several scaffolds [34]. Genes were annotated by NCBI, KEGG, and COG databases. For analysis of gene clusters encoding secondary metabolites, the antiSMASH 5.0 database was used [35]. The DNA G+C content of the strain was calculated from the genome sequence. The average amino acid identity (AAI) value (http://enveomics.ce.gatech.edu/aai/, accessed on 2 January 2021), the average nucleotide identity (ANI) value (http://enve-omics.ce.gatech.edu/ani/, accessed on 2 January 2021), the digital DNA-DNA hybridization (dDDH) value (http://ggdc.dsmz.de/distcalc2.php, accessed on 2 January 2021), and the percentage of conserved proteins (POCP) value [20] between two genomes were calculated to analyze relatedness between the isolate and the closely related strain N1E253^T^. A concatenated alignment of 120 ubiquitous single-copy genes of strain 5K15^T^ and the related strains was performed with GTDB-Tk v1.3.0 [36]. The phylogenetic tree based on these 120 ubiquitous genes was reconstructed by IQ-TREE [37] using the LG+F+I+G4 model and 1000 bootstrap replicates. Some other analysis methods referred to the previous published articles [38,39].

## 4. Conclusions

A new member of the phylum *Verrucomicrobiota,* strain 5K15^T^, was recovered from kelp taken from Li Island in China, being shown to be most closely related to the type strain *Oceaniferula marina* N1E253^T^. The characterization of strain 5K15^T^ using polyphasic methods suggests that it belongs to a new *Oceaniferula* species, which we named *Oceaniferula flavus* sp. nov. For general use, we propose the name *Oceaniferula flavus* due to the yellow color of the colonies. The genome sequence of the strain (genome size 3.95 Mbp) showed the presence of four putative biosynthetic gene clusters (BGCs), including two terpene biosynthetic gene clusters, one arylpolyene biosynthetic cluster, and one type III PKS cluster. Strain 5K15^T^ and *O. marina* N1E253^T^ contained 106 and 203 carbohydrate-active enzymes, including 17 and 13 carbohydrate esterases (CEs), 79 and 137 sulfatase encoded genes, 24 and 52 sulfatase-like hydrolase/transferase-encoded genes, and 25 and 36 arylsulfatase encoded genes, respectively, which are essential for dismantling the heavily decorated structure. The sulfated polysaccharides are the main components of seaweed and are challenging to degrade for bacteria compared to other monomers. The genus *Oceaniferula* of phylum *Verrucomicrobiota* provides candidates for the degradation of highly sulfated polysaccharides in the production of active metabolites from seaweeds. On the basis of the analysis of this research, we will perform experiments to confirm the degrading ability of genus *Oceaniferula* in the future. The structure and function of the seaweed degradation products by this genus will be illuminated deeply for the development of active drugs.

## Figures and Tables

**Table 1:** Differential characteristics of strain 5K15^T^ and the related type strain.

Characteristic	5K15^T^	*O. marina* N1E253^T^
Cell size (μm)	0.3–0.5 × 0.7–2.0	0.5–0.8 × 1.5–2.0 ^a^
Temperature range (°C)	15–37	15–37 ^a^
Growth pH	6.0–8.5	7.0–9.0 ^a^
Tolerance to NaCl (%)	1.0–5.5	1.5–5.0 ^a^
Catalase	–	+ ^a^
Oxidase	–	+ ^a^
Hydrolysis of starch	–	+ ^a^
API 20E test:		
Tryptophan deaminase	–	+
Voges–Proskauer reaction	+	+
Oxidation of (Biolog GEN III):		
d–Maltose	–	+
d-Mannose	+	–
d-Fructose	+	–
d-Raffinose	+	–
Enzyme activities (API ZYM):		
Leucine arylamidase	+	+
N-acetyl-β-glucosaminidase	–	+
Acid production (API 50CHB):		
d-Gentiobiose	–	+
d-Trehalose	–	+
l-Sorbitose	+	–
d-Lyxose	+	–
DNA G+C content (mol%)	55.4	52.0

All data are from this study unless otherwise indicated. +, positive; –, negative. Data from: ^a^ Jin et al. (2021) [13].

**Table 2:** Fatty acid compositions (%) of strain 5K15^T^ and the related strain.

Fatty Acid	5K15^T^	*O. marina* N1E253^T^
Branched fatty acids		
Iso-C_12:0_	6.7	-
Anteiso-C_13: 0_	1.4	-
Iso-C_14:0_	**28.5**	**33.3**
Anteiso-C_15: 0_	**11.8**	4.9
Iso-C_16:0_	9.5	7.3
Straight-chain fatty acids		
C_12:0_	Tr	1.2
C_14:0_	Tr	1.4
C_16:0_	**10.0**	**12.9**
C_17:0_	4.0	7.0
C_18:0_	Tr	1.3
Unsaturated fatty acids		
C_15: 1_*ω*6*c*	2.2	1.5
C_15: 1_*ω*8*c*	-	1.9
C_17: 1_*ω*6*c*	Tr	4.2
C_17: 1_*ω*8*c*	1.2	6.2
Hydroxy fatty acids		
C_12: 0_ 3-OH	2.0	Tr
Iso-C_12: 0_ 3-OH	2.3	-
Iso-C_14: 0_ 3-OH	Tr	3.8
Summed features *		
Summed feature 3	9.3	7.0

Tr, traces (<1.0%); -, not detected. Fatty acids amounting to (<1.0%) are not shown; dominant fatty acids (≥10.0%) are highlighted in bold. All data are from this study. * Summed features are groups of two or three fatty acids that are treated together for the purpose of evaluation in the MIDI system and include both peaks with discrete equivalent chain lengths (ECLs) as well as those where the ECLs are not reported separately. Summed feature 3 was listed as C16:1ω7c and/or C16:1ω6c.

**Table 3:** Genome statistics of strain 5K15^T^ and the related type strain.

Characteristic	5K15^T^	*O. marina* N1E253^T^
GenBank accession number	JAFBGL000000000	JACBAZ000000000
Genome size (bp)	3,953,077	5,073,947
N50 Value (bp)	346,557	785,939
Contigs (no.)	51	88
G+C content (mol/%)	55.4	52
**Annotation Results by the NCBI Prokaryotic Genome Annotation Pipeline (PGAP)**		
Genes (no.)	3354	3927
Protein-coding genes	3293	3855
tRNAs (no.)	45	46
rRNAs (no.)	3	7
ncRNAs (no.)	4	3

## Data Availability

The GenBank accession numbers for the 16S rRNA gene sequence and draft genome sequence of strain 5K15^T^ were MW260263 and JAFBGL000000000, respectively.

## Supplementary Materials

The following supporting information can be downloaded at https://www.mdpi.com/article/10.3390/md21010031/s1, Figure S1: Fast tree based on 16S rRNA gene sequences showing the phylogenetic position of strain 5K15^T^ among closely related taxa. Bootstrap values (expressed as percentages of 1000 replications) of >70% are shown at branch points. *Escherichia coli* JCM 1649^T^ (AB242910) was used as an outgroup. Figure S2: IQ tree based on 16S rRNA gene sequences showing the phylogenetic position of strain 5K15^T^ among closely related taxa. Bootstrap values (expressed as percentages of 1000 replications) of >70% are shown at branch points. *Escherichia coli* JCM 1649^T^ (AB242910) was used as an outgroup. Figure S3: Scanning electron microscopy of cells of strain 5K15^T^. Figure S4: Two-dimensional TLC of the polar lipids of strain 5K15^T^ and strain *O. marina* N1E253^T^ (a, 5K15^T^; b, N1E253^T^). Figure S5: The cluster of orthologous (COG) classifications of strain 5K15^T^ and strain *O. marina* N1E253^T^. Table S1: Comparison of secondary metabolites predicted by antiSMASH.
