# Supplementary material for: Description and Genomic Characterization of Oceaniferula flavus sp. nov., a Novel Potential Polysaccharide-Degrading Candidate of the Difficult-to-Cultivate Phylum Verrucomicrobiota Isolated from Seaweed
Source: Mar Drugs. 2022 Dec 29;21(1):31. doi: 10.3390/md21010031 (PMC9865893; doi:10.3390/md21010031)
Supplement: Supplementary file 1 [file marinedrugs-21-00031-s001.zip › marinedrugs-2079181-supplementary.pdf]

Supplementary Material

Description and Genomic Characterization of *Oceaniferula flavus* sp. nov., A Novel Potential Polysaccharide-Degrading Candidate of the Difficult-to-Cultivate Phylum Verrucomicrobiota Isolated from Seaweed

Meng-Qi Ye[1,2], Chuan-Bo Jin[1], Xin-Jiang Liu[1], Xin-Yun Tan[1], Yu-Qi Ye[1], and Zong-Jun Du[1,2,3]*
[1]Marine College, Shandong University, Weihai, Shandong, 264209, PR China
[2] Weihai Research Institute of Industrial Technology of Shandong University, Weihai, 264209, China
[3]State Key Laboratory of Microbial Technology, Shandong University, Qingdao, Shandong, 266237, PR China

***Author for correspondence:**

Zong-Jun Du, Email: duzongjun@sdu.edu.cn

Tel and Fax: +86-631-5688303

Supplementary Figure S1 Fast tree based on 16S rRNA gene sequences showing the phylogenetic position of strain 5K15^{T} among closely related taxa. Bootstrap values (expressed as percentages of 1000 replications) of >70 % are shown at branch points. *Escherichia coli* JCM 1649^{T} (AB242910) was used as an outgroup.

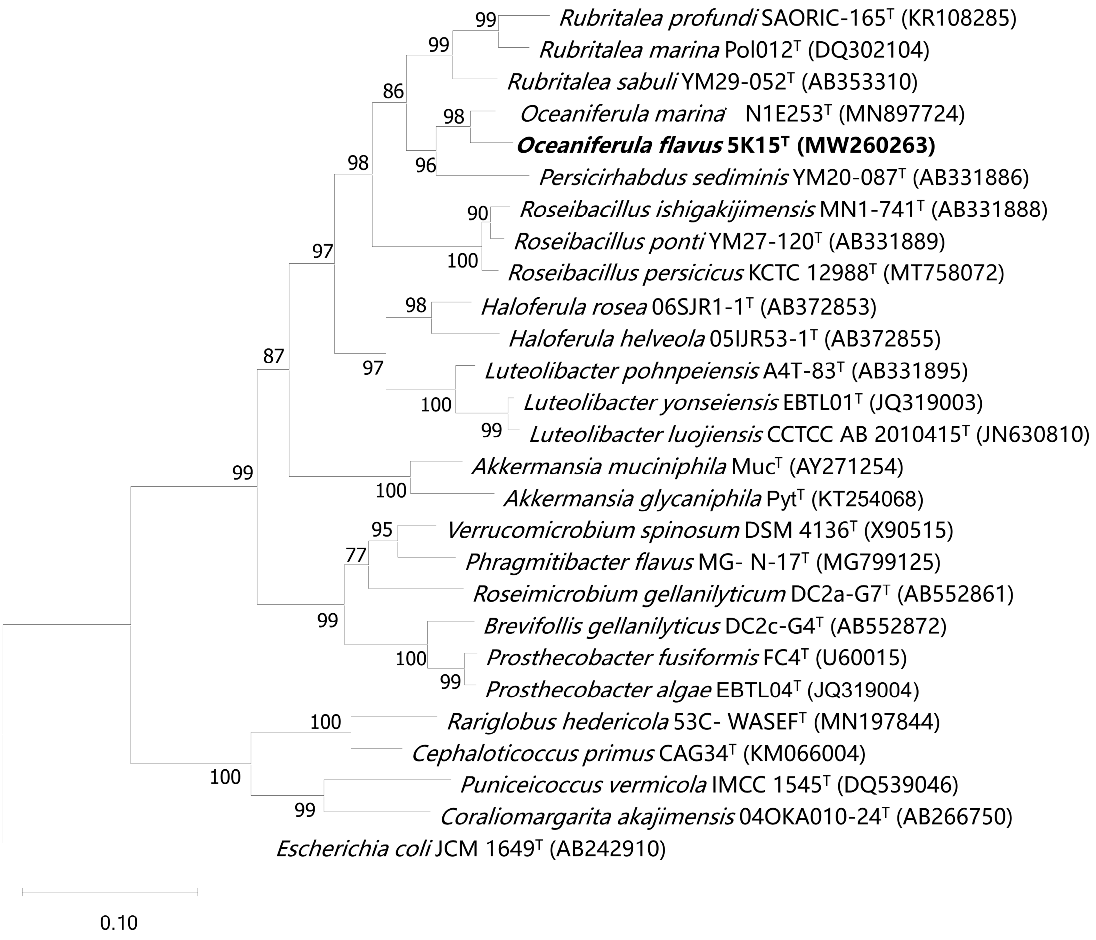

Supplementary Figure S2 IQ tree based on 16S rRNA gene sequences showing the phylogenetic position of strain 5K15^{T} among closely related taxa. Bootstrap values (expressed as percentages of 1000 replications) of >70 % are shown at branch points. *Escherichia coli* JCM 1649^{T} (AB242910) was used as an outgroup.

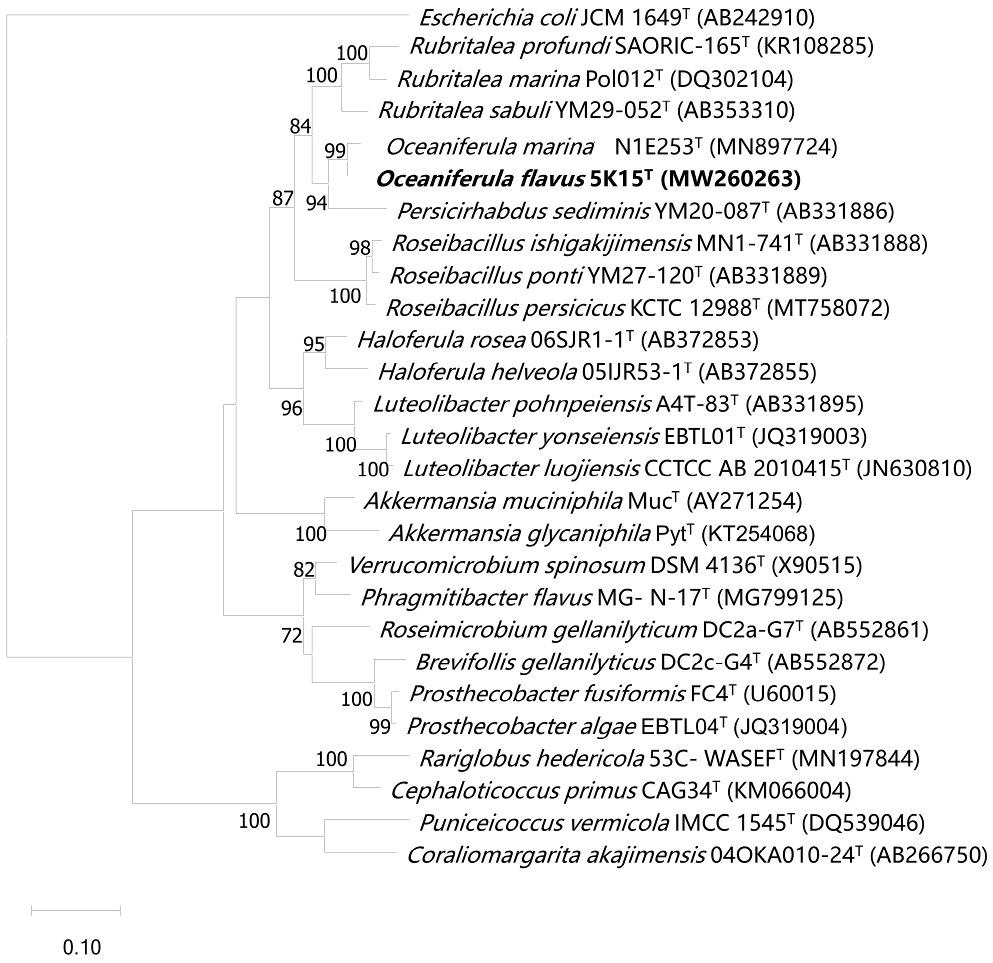

Supplementary Figure S3 Scanning electron microscopy of cells of strain 5K15^{T}. Cells were grown on 1/2-strength R2A agar (BD) with 75 % artificial seawater at 33 °C for 3 days.

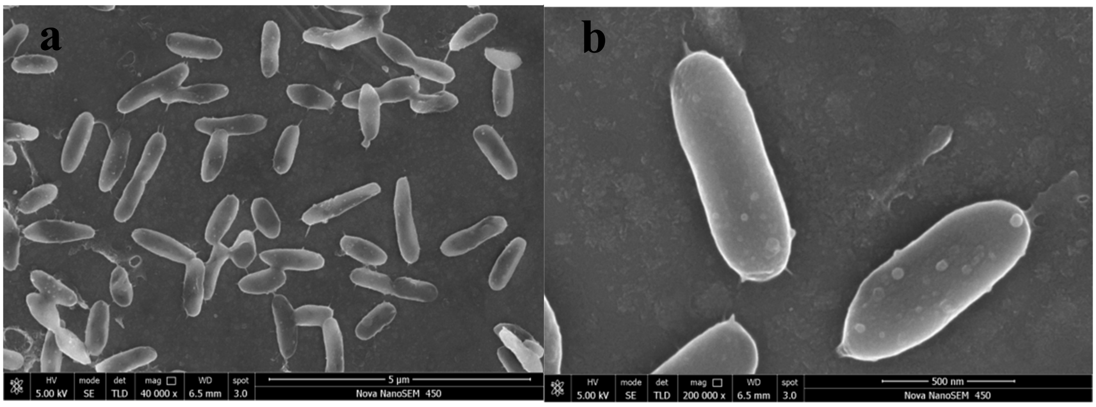

Supplementary Figure S4 Two-dimensional TLC of the polar lipids of strain 5K15^T and strain *O. marina* N1E253^T (a, 5K15^T; b, N1E253^T) . phosphatidylethanolamine(PE), diphosphatidylglycerol(DPG), unidentified phospholipid (PL), phosphatidylglycerol (PG), unidentified aminolipids (AL)， unidentified GlycoLipid (GL), unidentified polar lipids (L1, L2, L3)

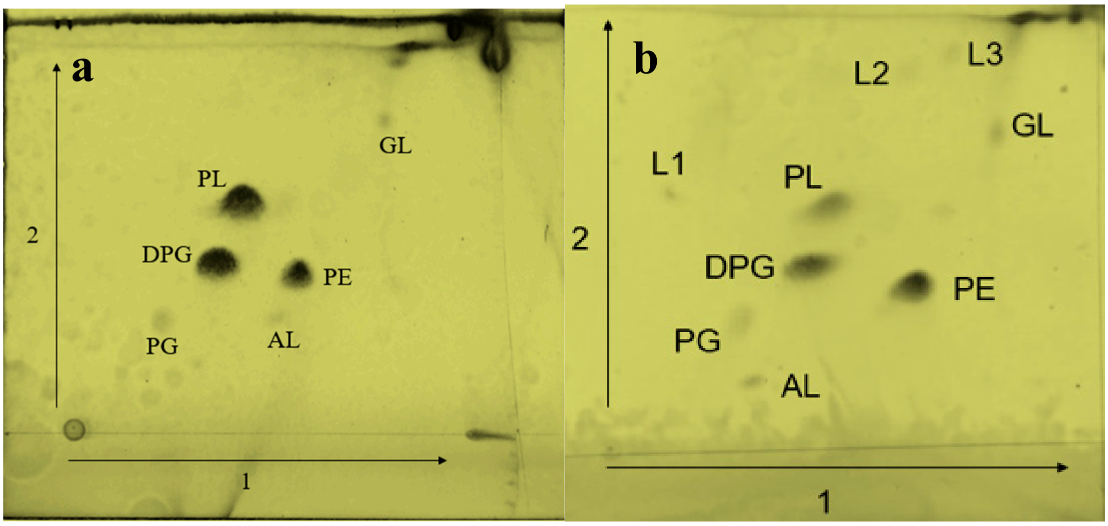

Supplementary Figure S5 The cluster of orthologous (COG) classifications of strain 5K15^T and strain *O. marina* N1E253^T

The y-axis indicates presents the different functional categories. The x-axis indicates the number of unigenes in a specific functional cluster.

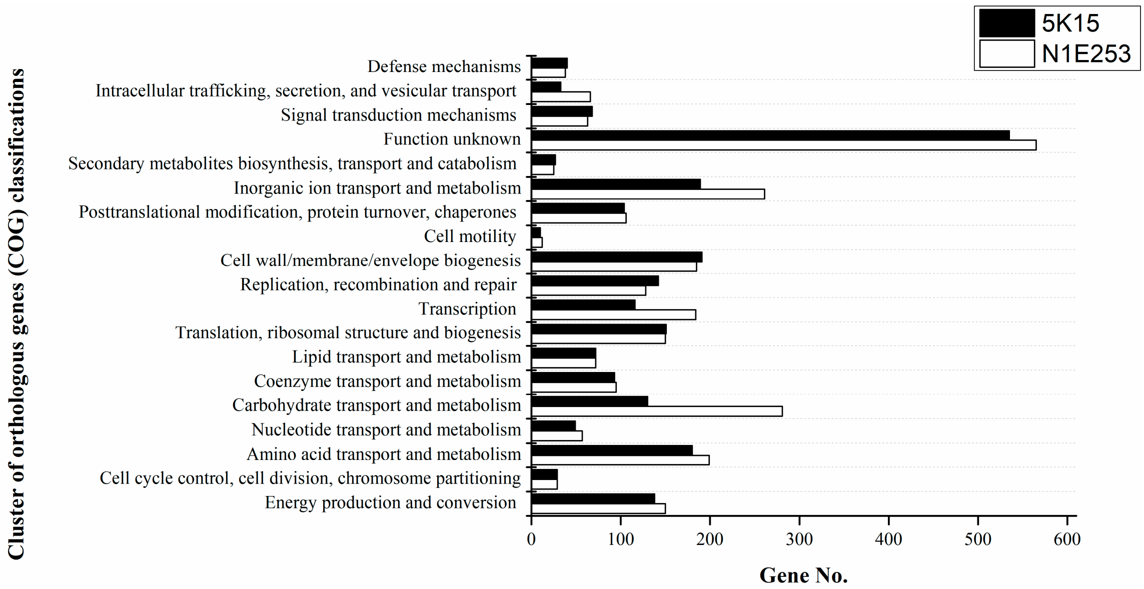

Supplementary Table S1 Comparison of secondary metabolites predicted by antiSMASH.

**Cluster**	**Region**	**Type**	**Location (bp)**	**Total length (bp)**	**Gene Count**
			$5K15^T$		
1	1.1	Terpene	679,788 - 700,765	20,978	18
2	22.1	Aryl polyene	260,229 - 301,398	41,170	41
3	25.1	Terpene	241,929 - 262,753	20,825	22
4	30.1	T3PKS	1 - 36,965	36,965	29
			O. marina $N1E253^T$		
1	11.1	Terpene	507,154 - 522,284	15,131	15
2	11.2	Terpene	1,115,405- 1,136,226	20,822	12
3	22.1	Phosphonate	342,728 - 379,214	36,487	35

T3PKS: Type III polyketide synthase cluster.
